# A Case of Poisoning by Ruta Graveolens

**Published:** 1853-11

**Authors:** George F. Cooper

**Affiliations:** Savannah, Geo.


*A Case of Poisoning by Ruta Graveolens.* By George F. Cooper, M. D., Savannah, Geo.—Isaac, a negro man, æt. 35, of good constitution and habits, had dysentery for six days, which was apparently confined to the rectum and for which he received mild treatment, without, however, any decided benefit. The disease seemed gradually to abate, and on Saturday, 14th Aug., '52, 7. A. M., he was entirely clear of any febrile excitement and only suffered slight tenesmus upon going to stool. He was so nearly recovered that I did not expect to visit him again. It seems he became dissatisfied with his slow rate of improvement and determined to expedite it if possible. He procured a handful of Rue, and after contusing it, he added about ℥iv of brandy, which he obtained from his owner, and after expressing it, took three drinks in pretty quick succession. It is proper to state he went after the brandy himself—forty or fifty yards—and his master regarding him as so nearly well, without hesitation let him have it. Violent symptoms were developed in the course of an hour, and I was sent for, but, being absent, did not reach him until about two o'clock, P.M., when I found him in the following condition : nausea and vomiting, violent abdominal pains and tenesmus, with frequent thin, bloody dejections, abdomen tender and considerably distended, troublesome irritation of the neck of the bladder giving rise to frequent and painful efforts at micturition. The fever was not of a high grade, skin being somewhat above the normal temperature and perspirable. The thirst was not very urgent, tongue clear, his pulse was from 90 to 95 and very irregular, his countenance expressive of much anxiety and he very restless, respiration but little hurried. Venesection was performed to the amount of 15 or 20 oz., or until the circulation was impressed. Large doses of opium—from grs. ij to grs. iij. in conjunction with the same quantity of Hydrarg. C. Creta and Acet. Plumb. were ordered every 2 or 3 hours. Mustard Cataplasms to the abdomen and diluent drinks. His abdomen was also cupped. Aug. 15. Not at all improved, except that he does not suffer so much pain as on yesterday. Dejections from his bowels continue undiminished in frequency and same in character and are exceedingly offensive. In the afternoon as there seemed to be no great degree of general excitement, at least not enough to forbid it, and as he appeared in no wise better, I gave him gr. ½ Nitrat. Argent. combined with gr. 1 Opium every 2 or 3 hours.

Continuing the former powders at prolonged intervals, 6 by 8 emplast. cantharid. was ordered upon the abdomen at intervals. 16th.—I found this morning that his stools were not so frequent nor offensive, their character otherwise remained apparently the same. Pulse still very irregular, from 90 to 95, with rather a better volume than before; in other respects in statu quo. In the afternoon, as he appeared partially narcotized, the above treatment was withheld and he placed upon Turpentine emulsion and Sol. Chlorid. Sod. He now became so annoyed with irritable stomach and real convulsive hiccough that it was with great difficulty that he could retain any article of diet or medicine, the presence of the slightest amount of either gave rise to the latter in a most distressing form. Nothing done had any good effect, he continued to sink and died on the 19th. I would remark, I have never seen an instance where the stools were as frequent, and in no case have I observed the powers of nature to resist so long such a strain. For the first two or three days two vessels had to be used, one could not be emptied and returned with sufficient dispatch for his use. It is to be regretted that an autopsy was not made. The case, I presume will speak for itself, and its cause I apprehend will not be questioned. This article (Rue) has been used to procure abortion, and the effects as recorded by other observers seem to quadrate with those laid down above.—*Nashville Journal of Medicine and Surgery.*

